# Supplementary figures and images for: One-Pot Preparation of Mixed-Mode Reversed-Phase Anion-Exchange Silica Sorbent and its Application in the Detection of Cyclopiazonic Acid in Feeds and Agricultural Products
Source: Foods. 2024 May 12;13(10):1499. doi: 10.3390/foods13101499 (PMC11119939; doi:10.3390/foods13101499)

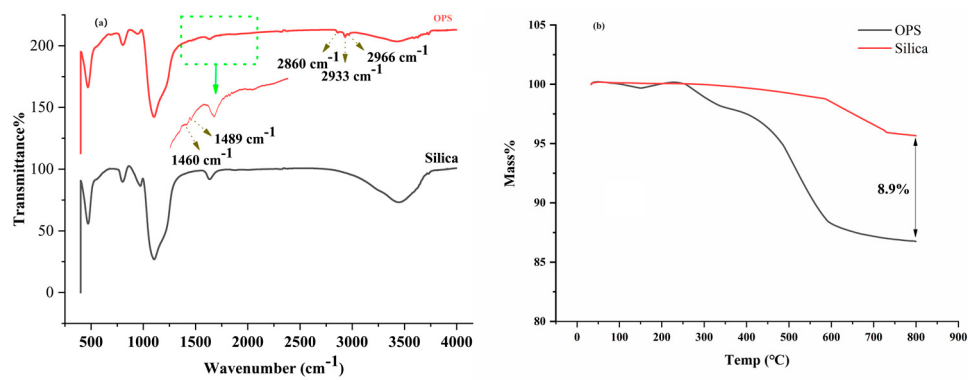

**Figure S1.** FTIR spectra (a) and TGA curves (b) of mixed-mode OPS sorbent and pure silica.

Supplement: Supplementary file 1 [file foods-13-01499-s001.zip › foods-2976532-supplementary.pdf]
